# Fatty Acid Profiling of Fish Fin Tissues: Implications for Non‐Lethal Sampling Methods

**DOI:** 10.1002/ece3.73784

**Published:** 2026-06-04

**Authors:** Lauren Comeau, Attiq Rehman, Bruce Phillips, Jill Hay, Andrew Swanson, Kurt M. Samways

**Affiliations:** ^1^ Department of Biological Sciences University of New Brunswick Saint John New Brunswick Canada; ^2^ Research and Productivity Council Fredericton New Brunswick Canada; ^3^ Cooke Aquaculture Inc. Blacks Harbour New Brunswick Canada

**Keywords:** Atlantic salmon, conservation, fatty acids, fish fin tissue, lipids, non‐lethal

## Abstract

Fatty acid (FA) profiling is crucial for understanding fish health and diet. However, the critical status of many fish species makes lethal sampling problematic and/or prohibited. This study evaluates the potential of fish fin tissues as non‐lethal alternatives to muscle tissue for FA profiling using Atlantic Salmon ( *Salmo salar* ). Lipid extractions from the muscle, adipose fin, inner‐caudal fin, and outer‐caudal fin tissues of 11 individuals were analyzed using a modified version of the Mojonnier method. Linear regressions for FA composition and concentration revealed a strong relationship between adipose fin and muscle tissues (*R* ^2^ = 0.98, *p* < 0.001 and *R* ^2^ = 0.98, *p* < 0.001; respectively). The relationships between outer‐caudal fin and muscle tissue for either FA composition and concentration were also significant (*R* ^2^ = 0.64, *p* < 0.001 and *R* ^2^ = 0.62, *p* < 0.001, respectively). Both outer‐caudal and adipose fin tissues had lower intra‐tissue variability than muscle tissue. This study suggests that adipose fin tissue may provide a suitable non‐lethal alternative for FA profiling in fish, given its strong correlation with muscle tissue, minimal intra‐tissue variation, and consistency in FA composition and concentration. Despite exhibiting distinct FA profiles relative to muscle tissue, outer‐caudal fin tissue showed consistent proportional relationships that may support its application in non‐lethal FA studies where adipose fins are unavailable. Non‐lethal FA sampling methods support research and conservation efforts and offer an ecologically responsible tool for the study and protection of fish populations worldwide.

## Graphical abstract

Fatty acid (FA) profiling is vital for assessing fish health, diet, food web functioning, yet lethal sampling poses ethical and conservation challenges. This study demonstrates that adipose and outer‐caudal fin tissues strongly correlate with muscle tissue in FA composition and concentration, offering reliable non‐lethal alternatives. These findings support ecologically responsible approaches to fish health monitoring and conservation research.

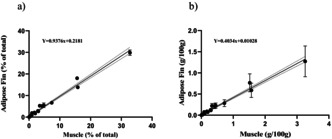

## 1 Introduction

Globally, fish biodiversity is facing unprecedented threats, with numerous species now classified as threatened, endangered, or extinct (Millennium Ecosystem Assessment 2005). Key pressures such as habitat loss, overexploitation, invasive species, climate change, among others, have placed significant pressure on freshwater and marine fish populations (Arthington et al. 2016). These stressors not only contribute to population declines but also disrupt trophic dynamics and reduce the availability of critical nutritional resources (Donohue et al. 2017). In response, conservation efforts have intensified to safeguard fish populations and restore ecosystem function (Reid et al. 2019).

Lipids, particularly fatty acids (FAs), are fundamental to the energy dynamics and physiological health of aquatic organisms (Kainz and Fisk 2009). As essential compounds, lipids improve the nutritional status of aquatic food webs and the health and fitness of aquatic organisms (Samways et al. 2017; Kainz and Fisk 2009). FA profiling is a valuable and widely adopted tool for assessing the fish health (Arts and Kohler 2009), conservation and trophic ecology, dietary structure (Budge et al. 2006; Samways et al. 2017; Twining et al. 2020; Xu et al. 2020), and predator–prey relationships (Iverson et al. 2004; Parrish et al. 2015; Péron et al. 2023). In aquaculture and wild populations alike, FA analyses inform assessments of immune response, cardiac function, stress physiology, and nutritional and energy availability. All factors which are critical to conservation management and stock recovery.

Traditionally, FA profiling in fish requires lethal sampling to obtain tissues such as liver or muscle (Miller et al. 2006), which poses ethical, legal, and logistical challenges when dealing with imperiled species. Consequently, the imperative to reduce harm has prompted the development of non‐lethal sampling techniques as viable alternatives and is expected to become integral to sampling programs going forward (Jeffries et al. 2021).

Previous studies have explored the use of adipose fin tissue as a non‐lethal alternative for FA analyses in fish. For example, Olsen et al. (2013) demonstrated that FA profiles derived from adipose fin tissue in Atlantic salmon could reflect dietary history when comparing triacylglycerol (TAG) and total lipid (TOT) FA composition. Similarly, Madhun et al. (2017) used adipose fin FA analyses to characterize the ecological profiles of escaped farmed Atlantic salmon entering the River Etne, successfully identifying individuals that had escaped aquaculture facilities at different stages of production. Together, these studies provide growing evidence supporting the utility of non‐lethal FA sampling approaches in fish. Emerging research has also identified adipose and caudal fin tissues as viable non‐lethal alternatives for physiological and molecular analyses (Thorstensen et al. 2022). However, despite the minimally invasive nature of caudal fin sampling, its suitability for FA profiling remains poorly understood. Furthermore, previous studies have largely focused on FA composition alone, while direct comparisons between FA composition and concentration across multiple non‐lethally sampled tissues have yet to be thoroughly evaluated.

Atlantic salmon ( *Salmo salar* ) are a culturally, economically, and ecologically important species, which exemplifies the urgent need for minimally invasive research techniques. Once widespread across the North Atlantic, many populations are now in severe decline due to a combination of anthropogenic threats (COSEWIC 2006; Thorstad et al. 2021). In Canada, Atlantic salmon are not only ecologically important but hold profound cultural value and play a vital role in many traditional practices for Indigenous communities across eastern Canada (Gross 1998). FA analysis has been used to investigate Atlantic salmon trophic ecology, stock structure, conservation measures, and the impact of aquaculture escapes (Skilbrei et al. 2015; Samways et al. 2017; Parzanini et al. 2020). Atlantic salmon represent an ideal model species for evaluating the effectiveness of non‐lethal FA sampling methods.

In this study, we evaluate whether adipose and caudal fin tissues can serve as reliable proxies for muscle tissue in FA analysis or additional tissue options for non‐lethal FA sampling. Specifically, we compare the composition and concentration of FAs across these tissues to assess the feasibility of using non‐lethal samples for nutritional and ecological monitoring. Previous studies have largely focused on single tissue comparisons or FA composition alone, leaving uncertainty regarding how different non‐lethal tissues compare across both compositional and concentration‐based analyses and whether proportional relationships are consistent with multivariate similarity metrics. By developing and validating alternative sampling methods, this research aims to reduce harm to vulnerable fish populations while advancing the application of FA profiling in conservation science.

## 2 Methods

### 2.1 Sample Collection

In August 2024, this study opportunistically obtained Atlantic salmon tissue samples that had been previously sacrificed at the Cooke Aquaculture commercial processing facility in St. George, New Brunswick, Canada. No live animals were sacrificed for the sole purposes of this study; therefore, ethical approval was not required. All fish originated from a commercial aquaculture operation; therefore, they received the same diet and were of the same age range, relative size, and genetic family.

**FIGURE 1:**
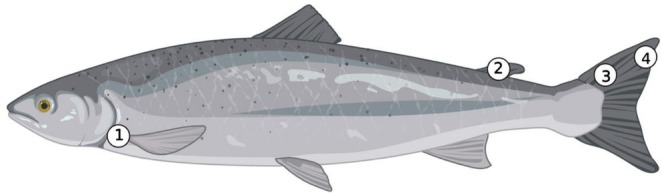
Tissue sample locations on Atlantic salmon ( *Salmo salar* ) included: (1) Muscle, (2) Adipose fin, (3) Inner caudal fin, (4) Outer caudal fin.

A total of 12 fish were haphazardly selected and had a muscle sample taken from the area immediately anterior to the operculum (Figure 1). Equipment was cleaned between individuals. The number of individuals chosen for this project was based on a power analysis on the FA profiles of a proxy sample of juvenile wild Atlantic salmon, as well as the availability of intact individuals, defined as those from which all required tissues originated from the same specimen. The muscle sample location and size were based solely on the availability of tissue remaining post processing. In addition, the adipose fin and the entire caudal fin were collected from the same individual using fish processing knives used at the facility (Figure 1). Although in the wild, the whole adipose fin and/or caudal fin would not be sampled, since these fish were already sacrificed, whole fins were collected. As the entire caudal fin was made available during sample collection, the inner and outer sections of the caudal fin were opportunistically selected as sampling locations to better understand FA differences along the length of the caudal fin (Figure 1). All tissues were stored in a laboratory freezer to preserve the integrity of the biological samples.

### 2.2 Lipid Extraction

Lipid extractions were conducted at the Research and Productivity Council (RPC) facility in Fredericton, New Brunswick. Lipids were extracted from three replicate samples of each tissue (muscle, adipose fin, inner‐caudal and outer‐caudal fins) from 12 individual Atlantic salmon (48 samples in total) using a modified version of the Mojonnier method (RPC 2024; Hooi et al. 2004; Nielsen 2024). One gram of each tissue sample was homogenized and transferred to Mojonnier flasks, where 100 mg of pyrogallic acid and 200 μL of glycerol triundecanoate internal standard were added to all samples. Next, 2 mL of ethanol and 10 mL of 100% HCl were added to all flasks, which were then placed in a 75°C hot water bath for complete digestion. Flasks were then removed and cooled with the addition of 10 mL of ethanol. Once at room temperature, the lipids were separated from the mixture using three separate additions of ethyl ether and petroleum ether. The top, clear layer, which contained the lipids, was decanted into labeled beakers for each sample. Beakers with the lipid fractions were then placed in a 53°C hot water bath to facilitate the evaporation of ethers from the lipids. Beakers were then removed from the hot water bath and the remaining fat was transferred to culture tubes using petroleum ether rinses. Under a stream of nitrogen, the petroleum ether was evaporated, and 2 mL of 1% sulfuric acid in methanol and 1 mL of toluene were added. Samples were stored in a 50°C oven overnight to allow for methylation. After approximately 18 h, the samples were cooled to room temperature, and 5 mL of water, along with 2 mL of hexane, were added to each sample. The upper layer, consisting of the lipids, was transferred to new culture tubes containing sodium sulfate, using hexane rinses. Each sample was then transferred to new culture tubes and diluted to 10 mL with hexane. Finally, 1.5 mL of the solution was prepared in vials for gas chromatography. The FA methyl esters were analyzed using an Agilent Technologies 7890B Gas Chromatography system with Auto‐Sampler 7693 (Agilent Technologies, Santa Clara, California, USA) at RPC. Glycerol trindecanoate internal standard, added during lipid extraction, was used to determine the relative concentration of FA peaks. Due to an error during gas chromatography analysis, the results for all tissues from one individual were excluded from all statistical analyses, resulting in a final sample size of *n* = 11.

### 2.3 Statistical Analysis

A Principal Component Analysis (PCA) was conducted to identify the similarities in FA composition (% of total) and concentration (g/100 g) between tissues and to determine which FAs accounted for major sources of variation within the data; therefore, minimizing redundancy. The Euclidean distance for each sample to the group centroid was calculated to provide a metric of FA profile variability within each tissue (Layman et al. 2007). The centroid was defined as the mean PC1 and PC2 values for all samples in each tissue. The Euclidean distances (amount of variation) were compared between tissues using an analysis of variance (ANOVA). Euclidean distances in PCA ordination space were used as an exploratory measure of relative within‐tissue variability among samples. Because PCA axes do not represent total multivariate variance, these comparisons were interpreted qualitatively rather than as definitive measures of overall variability.

Using the statistical software PRIMER, FA composition and concentration were compared between and within each tissue. FA profiles were analyzed with a permutational multivariate analysis of variance (PERMANOVA) based on a Bray–Curtis dissimilarity matrix, with data square‐root‐transformed to meet ANOVA assumptions and tested for homogeneity of variance. In the PERMANOVA, Tissue (four levels: muscle, adipose, inner caudal, and outer caudal) was the fixed factor, while Sample (the error term) was the random factor. Similarity percentage (SIMPER) analysis, which measures the top 90% of contributing variables, was used to calculate the average sample dissimilarity of each FA between and within tissues. Additionally, a linear regression was performed to assess the strength and nature of the relationship between each tissue and muscle.

## 3 Results

### 3.1 Principal Component Analyses

**FIGURE 2:**
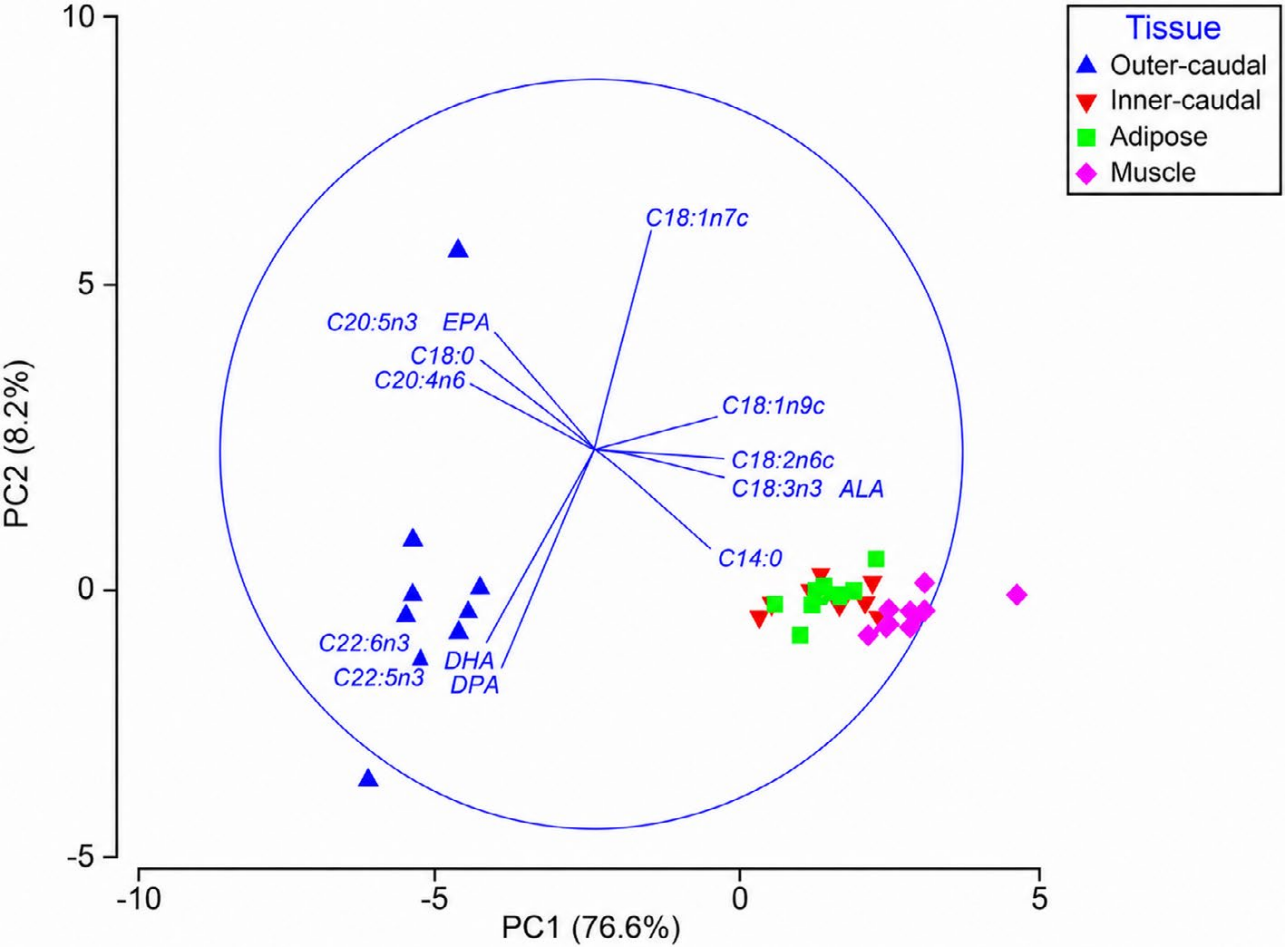
Principle component analysis (PCA) of muscle (pink diamonds), adipose (green squares), inner‐caudal (red inverted triangles) and outer‐caudal (blue triangles) fatty acid composition (% of total). The eigenvectors (represented by individual fatty acids and corresponding vector length) illustrate the importance of that fatty acid's contribution to the tissue on the first two principal component axes.

Principal component analyses (PCA) of FA profiles suggested distinct compositional differences among tissue types. When expressed as percentage of total FA, PCA ordination showed a strong separation along the first principal component axis (PC1), which accounted for 76.6% of the total variance (Figure 2). This axis clearly distinguished the outer‐caudal tissue from other tissue types. FAs contributing most strongly to this separation included 18:2n6, 18:3n3, and 18:1n9 (positive loadings), as well as 18:0, 20:4n6, and 22:6n3 (negative loadings). The second principal component axis (PC2) accounted for an additional 8.2% of variance, with 18:1n7 and 20:5n3 showing strong positive loadings, and 22:5n3 and 22:6n3 showing strong negative loadings.

These patterns suggest a distinct FA composition in the outer‐caudal fin tissue relative to the inner‐caudal fin, adipose fin, and muscle tissues, which exhibit considerable overlap (Figure 2). The outer‐caudal fin tissue was characterized by a higher relative abundance of long‐chain polyunsaturated FAs (i.e., C16:0, C18:0, C20:4n6, C20:5n3, C22:5n3 and C22:6n3) while the inner‐caudal fin, adipose fin, and muscle tissues were characterized by elevated proportions of shorter‐chain and monounsaturated FAs, including C14:0, C16:1n7, C18:1n9, C18:2n6, C18:3n3 and C20:2n6.

**FIGURE 3:**
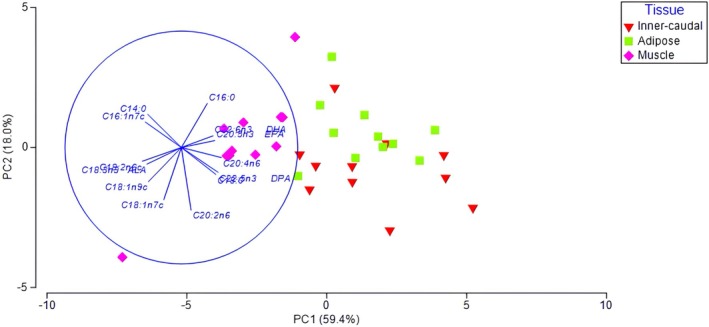
Principle component analysis (PCA) of muscle (pink diamonds), adipose (green squares), and inner‐caudal (red inverted triangles) fatty acid composition (% of total). The eigenvectors (represented by individual fatty acids and corresponding vector length) illustrate the importance of that fatty acid's contribution to the tissue on the first two principal component axes.

Additionally, a PCA based on the FA composition of inner‐caudal fin, adipose fin, and muscle tissue was conducted following the exclusion of outer‐caudal fin tissue. Because outer‐caudal tissue accounted for a large proportion of the variance in the initial compositional PCA, it was excluded to better evaluate patterns among the remaining tissues. PCA ordination showed separation along the first principal component axis (PC1), which accounted for 59.3% of the total variance (Figure 3). This axis primarily distinguished muscle tissue from adipose and inner‐caudal fin tissues, although the degree of separation was less pronounced than that observed previously for outer‐caudal tissue. FAs contributing most strongly to this separation included C18:2n6, C18:3n3, C18:1n9, and C16:1n7 (positive loadings), as well as C20:5n3, C22:6n3, C20:4n6, and C22:5n3 (negative loadings). The second principal component axis (PC2) accounted for an additional 18.2% of the total variance and was primarily associated with C16:0, C14:0, and C16:1n7 (positive loadings), while C20:2n6 and C18:1n7 exhibited strong negative loadings.

These patterns suggest moderate compositional separation between muscle tissue and the adipose and inner‐caudal fin tissues; however, the degree of separation was substantially lower than that observed in Figure 2. Muscle tissue was characterized primarily by elevated proportions of monounsaturated FAs, including C16:1n7, C18:1n9, and C18:1n7, whereas adipose and inner‐caudal fin tissues exhibited comparatively greater diversity in overall FA composition.

**FIGURE 4:**
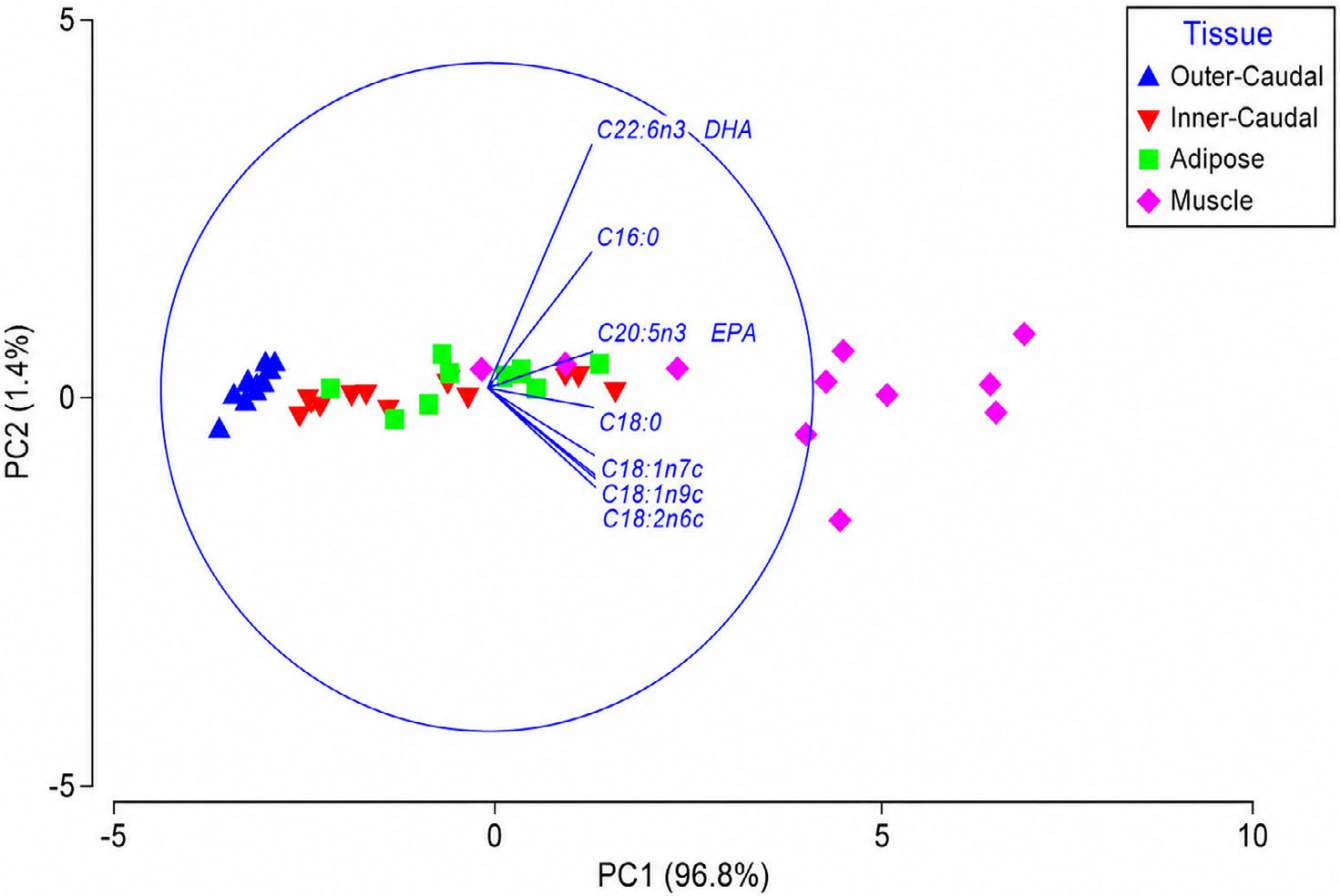
Principle component analysis (PCA) of muscle (pink diamonds), adipose (green squares), inner‐caudal (red inverted triangles) and outer‐caudal (blue triangles) fatty acid abundance (g/100 g). The eigenvectors (represented by individual fatty acids and corresponding vector length) illustrate the importance of that fatty acid's contribution to the tissue on the first two principal component axes.

When FA data were analyzed based on concentration (g/100 g), the first principal component axis (PC1) explained 96.8% of the total variance, with positive loading demonstrating the greatest differentiation between tissues (Figure 4). The second component (PC2) accounted for 1.4% of the variance and was driven by 22:6n3 (positive loading) and 14:0 (negative loading). Outer‐caudal fin tissue was characterized by negative PC1 scores, indicating relatively low FA concentrations, whereas muscle tissue exhibited the highest PC1 scores, indicating elevated total FA content and greater inter‐individual variability (Figure 4).

### 3.2 Euclidian Distance

**FIGURE 5:**
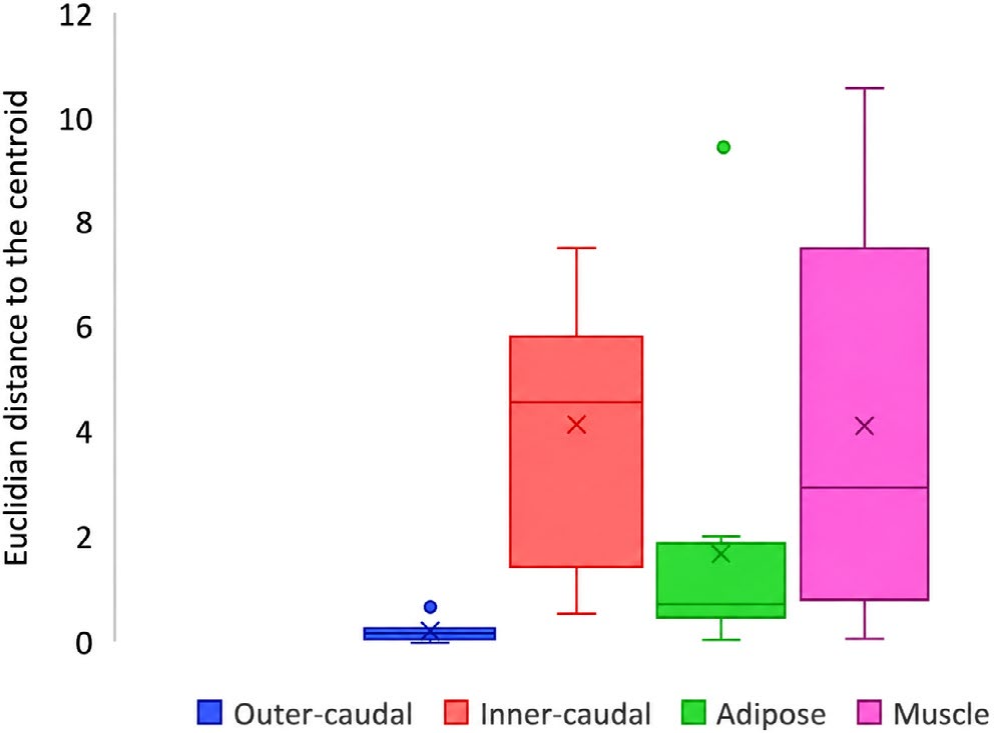
Box and whisker plot of the Euclidean distance to the centroid for fatty acid composition (% of total) for each tissue; outer‐caudal, inner‐caudal, adipose, and muscle.

FA composition differences, quantified as the median Euclidean distance to group centroid, suggest variability among tissue types (*F*_3,40_ = 6.29, *p* = 0.001). The outer‐caudal fin tissue exhibited the lowest within‐group variability (mean Euclidian distance = 0.24), while inner‐caudal fin tissue showed the greatest variation (mean Euclidian distance = 4.02) (Figure 5). Pairwise comparisons suggested relative differences in variability among all tissue types, although no clear differences were observed between inner‐caudal and muscle tissues (*t*_10_ = 0.02, *p* = 0.490) (Figure 5).

**FIGURE 6:**
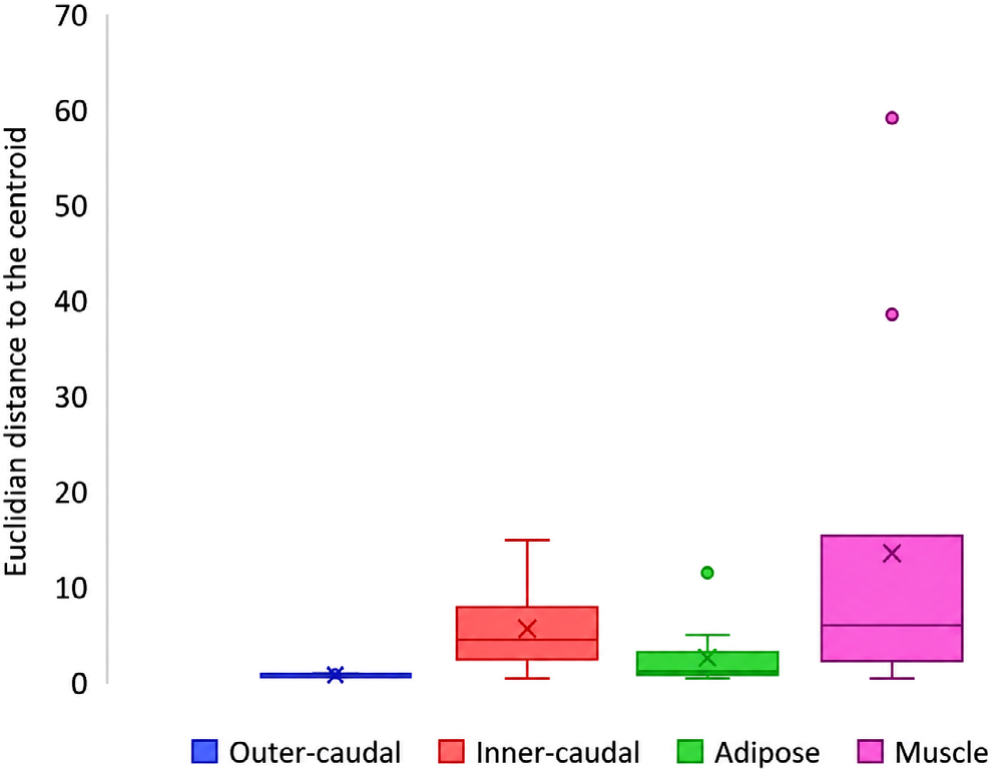
Box and whisker plot of the Euclidean distance to the centroid fatty acid concentration (g/100 g) for each tissue; outer‐caudal, inner‐caudal, adipose, and muscle.

Similar patterns were observed when assessing variation in FA concentration (g/100 g tissue). Euclidian distances to the centroid were lowest in the outer‐caudal fin tissue (mean Euclidian distance = 0.11) and greatest in muscle tissue (mean Euclidian distance = 12.14) (Figure 6). Differences in relative FA concentration variability among tissue types were suggested (*F*_3,40_ = 4.15, *p* = 0.010). No significant differences in variability were detected between inner‐caudal and adipose fin tissues (*t*_10_ = 1.70, *p* = 0.060), or between inner‐caudal fin and muscle tissue (*t*_10_ = −1.42, *p* = 0.090). All other pairwise comparisons showed significant differences (Figure 6).

### 3.3 Permutational Analysis of Variance

A total of 29 interrelated FAs were identified across all tissue types and quantified for both FA composition (percentage of total FAs; Table 1) and concentration (g/100 g tissue; Table 2). All identified FAs were included to minimize potential limitations. FA composition varied significantly among tissues (PERMANOVA; *F*_3,40_ = 19.69, *p <* 0.001). Pairwise comparisons revealed significant differences between most tissue types, except between inner‐caudal and adipose fin tissues (*t*_20_ = 6.43, *p* = 0.130), indicating a degree of compositional similarity. The greatest dissimilarity was observed between outer‐caudal fin and muscle tissues (*t*_20_ = 6.12, *p* < 0.001), while comparisons between inner‐caudal fin and muscle tissues (*t*_20_ = 2.81, *p* < 0.001) and adipose fin and muscle tissue (*t*_20_ = 2.36, *p* = 0.001) revealed smaller, yet significant differences (Table 1). Additional significant contrasts included outer‐caudal versus inner‐caudal fin tissues (*t*_20_ = 4.70, *p* < 0.001) and outer‐caudal versus adipose fin tissues (*t*_20_ = 5.50, *p* < 0.001; Table 1).

**TABLE 1:** Fatty acid percent composition of muscle, adipose fin, inner‐caudal and outer‐caudal tissue samples.

FAs	Muscle (±SD)	Adipose (±SD)	Inner‐Caudal (±SD)	Outer‐Caudal (±SD)
∑Saturates	23.47 ± 2.03	26.23 ± 1.35	25.13 ± 1.44	34.36 ± 5.51
C14:0	3.04 ± 0.23	2.69 ± 0.28	2.42 ± 0.31	0.97 ± 0.65
C15:0	0.23 ± 0.02	0.19 ± 0.10	0.10 ± 0.12	nd
C16:0	15.63 ± 2.40	18.02 ± 0.58	17.03 ± 0.72	23.12 ± 5.55
C17:0	0.21 ± 0.01	0.17 ± 0.11	0.10 ± 0.12	nd
C18:0	4.25 ± 0.27	5.12 ± 0.36	5.44 ± 0.78	10.27 ± 1.40
C20:0	0.11 ± 0.06	0.04 ± 0.07	0.04 ± 0.07	nd
∑Monounsaturates	44.12 ± 0.41	40.26 ± 0.20	40.48 ± 0.18	26.88 ± 0.85
C14:1n5c	0.04 ± 0.05	nd	nd	nd
C16:1n7c	7.39 ± 0.41	6.66 ± 0.50	6.38 ± 0.68	2.67 ± 0.39
C18:1	0.31 ± 0.04	0.22 ± 0.12	0.10 ± 0.12	nd
C18:1n9c	32.81 ± 1.87	29.85 ± 1.23	30.62 ± 1.02	20.45 ± 2.44
C18:1n7c	2.89 ± 0.15	2.86 ± 0.10	2.87 ± 0.11	2.43 ± 0.90
C20:1	0.34 ± 0.05	0.27 ± 0.10	0.17 ± 0.16	nd
C22:1n11	0.02 ± 0.04	nd	nd	nd
C22:1n9	0.14 ± 0.05	0.06 ± 0.08	0.04 ± 0.08	nd
C24:1n9	0.18 ± 0.04	0.34 ± 0.16	0.30 ± 0.22	1.33 ± 0.88
∑Polyunsaturates^a^	39.17 ± 0.05	32.56 ± 0.25	33.40 ± 0.06	38.64 ± 8.66
∑Omega‐6	26.39 ± 0.17	17.41 ± 0.16	18.04 ± 0.15	15.54 ± 0.59
C18:2n6c	15.86 ± 0.96	13.80 ± 0.75	14.17 ± 0.67	6.41 ± 0.88
C18:3n6	0.28 ± 0.04	0.25 ± 0.09	0.17 ± 0.13	nd
C20:2n6	0.90 ± 0.06	0.92 ± 0.05	0.96 ± 0.05	nd
C20:3n6	0.49 ± 0.10	0.57 ± 0.15	0.68 ± 0.28	1.22 ± 1.13
C20:4n6	7.91 ± 1.15	1.85 ± 0.57	2.05 ± 0.57	7.91 ± 1.15
C22:2n6	0.95 ± 0.12	0.02 ± 0.03	0.01 ± 0.04	nd
∑Omega‐3	12.78 ± 0.27	15.15 ± 0.69	15.36 ± 0.30	23.1 ± 4.12
C18:3n3 ALA	2.02 ± 0.16	1.68 ± 0.12	1.78 ± 0.20	0.14 ± 0.24
C18:4n3	0.58 ± 0.05	0.55 ± 0.05	0.40 ± 0.26	nd
C20:3n3	0.11 ± 0.07	0.06 ± 0.08	0.06 ± 0.09	nd
C20:5n3 EPA	3.47 ± 0.50	5.26 ± 0.72	5.15 ± 0.88	7.69 ± 1.72
C22:5n3 DPA	1.79 ± 0.15	2.13 ± 0.27	2.28 ± 0.35	3.31 ± 1.14
C22:6n3 DHA	4.806 ± 1.10	5.47 ± 01.80	5.69 ± 0.82	11.96 ± 4.37
(n‐3/n‐6)	0.48	0.87	0.85	1.49
20:5n3/22:6n3	0.72	0.96	0.91	0.64
20:4n6/22:6n3	1.65	0.34	0.36	0.66

*Note:* Values (mean ± SD) are percentages of total FAs (nd = not detected). SD of ∑ calculated using pooled standard deviation.

^a^ Totals include Omega‐6 and Omega‐3 fatty acids.

**TABLE 2:** Fatty acid concentrations of muscle, adipose fin, inner‐caudal and outer‐caudal tissue samples.

FAs	Muscle (±SD)	Adipose (±SD)	Inner‐Caudal (±SD)	Outer‐Caudal (±SD)
∑Saturates	2.29 ± 0.03	1.12 ± 0.01	0.903 ± 0.01	0.28 ± 0.001
C14:0	0.30 ± 0.08	0.11 ± 0.03	0.09 ± 0.05	0.008 ± 0.006
C15:0	0.02 ± 0.006	0.009 ± 0.005	0.005 ± 0.006	nd
C16:0	1.52 ± 0.46	0.77 ± 0.21	0.61 ± 0.31	0.19 ± 0.08
C17:0	0.02 ± 0.006	0.008 ± 0.005	0.005 ± 0.006	nd
C18:0	0.42 ± 0.12	0.22 ± 0.06	0.19 ± 0.08	0.08 ± 0.02
C20:0	0.01 ± 0.006	0.002 ± 0.003	0.003 ± 0.004	nd
∑Monounsaturates	4.37 ± 0.12	1.70 ± 0.02	1.48 ± 0.04	0.21 ± 0.0002
C14:1n5c	0.005 ± 0.005	nd	nd	nd
C16:1n7c	0.72 ± 0.20	0.28 ± 0.08	0.24 ± 0.13	0.02 ± 0.004
C18:1	0.03 ± 0.01	0.01 ± 0.006	0.005 ± 0.007	nd
C18:1n9c	3.26 ± 1.01	1.27 ± 0.36	1.11 ± 0.59	0.16 ± 0.04
C18:1n7c	0.29 ± 0.09	0.12 ± 0.03	0.10 ± 0.05	0.02 ± 0.007
C20:1	0.03 ± 0.01	0.01 ± 0.004	0.008 ± 0.008	nd
C22:1n11	0.002	nd	nd	nd
C22:1n9	0.01 ± 0.008	0.003 ± 0.004	0.002 ± 0.004	nd
C24:1n9	0.02 ± 0.004	0.01 ± 0.007	0.01 ± 0.009	0.01 ± 0.008
∑Polyunsaturates^a^	3.08 ± 2.46	1.37 ± 0.47	1.20 ± 0.65	0.31 ± 0.42
∑Omega‐6	1.84 ± 0.04	0.74 ± 0.005	0.65 ± 0.01	0.12 ± 0.00005
C18:2n6c	1.57 ± 0.48	0.59 ± 0.17	0.52 ± 0.28	0.05 ± 0.01
C18:3n6	0.03 ± 0.008	0.01 ± 0.004	0.008 ± 0.007	nd
C20:2n6	0.09 ± 0.03	0.04 ± 0.01	0.03 ± 0.02	0.0005 ± 0.002
C20:3n6	0.05 ± 0.02	0.02 ± 0.007	0.02 ± 0.006	0.009 ± 0.009
C20:4n6	0.09 ± 0.02	0.08 ± 0.01	0.07 ± 0.02	0.06 ± 0.01
C22:2n6	0.009 ± 0.01	0.001 ± 0.003	0.0007 ± 0.002	nd
∑Omega‐3	1.24 ± 0.005	0.63 ± 0.001	0.54 ± 0.002	0.91 ± 0.0003
C18:3n3 ALA	0.20 ± 0.06	0.07 ± 0.02	0.07 ± 0.04	0.001 ± 0.002
C18:4n3	0.06 ± 0.02	0.02 ± 0.007	0.02 ± 0.01	nd
C20:3n3	0.01 ± 0.008	0.003 ± 0.004	0.004 ± 0.005	nd
C20:5n3 EPA	0.34 ± 0.10	0.22 ± 0.06	0.17 ± 0.07	0.06 ± 0.01
C22:5n3 DPA	0.18 ± 0.05	0.09 ± 0.02	0.08 ± 0.03	0.03 ± 0.01
C22:6n3 DHA	0.45 ± 0.11	0.23 ± 0.06	0.20 ± 0.08	0.10 ± 0.04
(n‐3/n‐6)	0.67	0.85	0.84	1.60
20:5n3/22:6n3	0.76	0.96	0.85	0.60
20:4n6/22:6n3	0.20	0.35	0.35	0.60

*Note:* Values (mean ± SD) are concentrations (g/100 g) of FAs (nd = not detected). SD of ∑ calculated using pooled standard deviation.

^a^ Totals include Omega‐6 and Omega‐3 fatty acids.

PERMANOVA based on FA concentration also identified significant differences among tissues (*F*_3,40_ = 25.66, *p* < 0.001; Table 2). All pairwise comparisons were statistically significant, except for inner‐caudal versus adipose fin tissues (*t*_20_ = 1.11, *p* = 0.272; Table 2). As with composition, outer‐caudal fin versus muscle tissue showed the greatest difference (*t*_20_ = 7.44, *p* < 0.001). Comparisons between inner‐caudal fin versus muscle tissue (*t*_20_ = 4.77, *p* < 0.001), adipose fin versus muscle tissue (*t*_20_ = 4.48, *p* < 0.001), outer‐caudal versus inner‐caudal fin tissue (*t*_20_ = 3.28, *p <* 0.001), and outer‐caudal versus adipose fin tissue (*t*_20_ = 5.25, *p* < 0.001) showed more moderate but statistically significant differences (Table 2).

FA composition varied among tissue types. In muscle, adipose fin, and inner‐caudal fin, the largest portion of total FAs was composed of monounsaturated FAs (MUFAs), predominantly 18:1n9, contributing 32.81%, 29.85% and 30.62%, respectively (Table 1). In contrast, outer‐caudal fin tissue composition was dominated by saturated fatty acids (SFAs) and polyunsaturated fatty acids (PUFAs), with 16:0 accounting for 23.12% of the total profile (Table 1). PUFA content in muscle, adipose, and inner‐caudal tissues was largely driven by omega‐6 FAs, contributing 26.39%, 17.41% and 18.04%, respectively (Table 1).

FA concentrations followed similar trends. MUFAs represented the highest concentrations in all tissues except outer‐caudal fin, primarily driven by 18:1n9, which was present at 3.26 g/100 in muscle, 1.27 g/100 g in adipose fin, and 1.11 g/100 g inner‐caudal fin tissues (Table 2). Inner‐caudal fin tissues had the highest concentrations of SFAs and PUFAs, with 16:0 reaching 0.19 g/100 g (Table 2). PUFA concentrations in muscle, adipose fin, and inner‐caudal fin tissues were primarily influenced by the omega‐6 FAs, contributing 1.84, 0.74, and 0.65 g/100 g, respectively (Table 2). In contrast, outer‐caudal fin tissue had a greater concentration of omega‐3 FAs (0.91 g/100 g) than omega‐6 FAs, further distinguishing its lipid profile from other tissues (Table 2).

### 3.4 Similarity Percentage Analysis (SIMPER)

**FIGURE 7:**
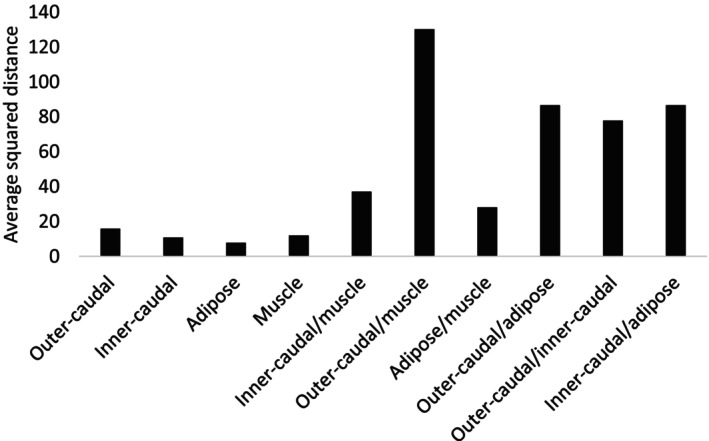
Within group average squared distance for each tissue, based on fatty acid composition (% of total), using average squared distance, as calculated using similarity percentage analysis.

A similarity percentage analysis (SIMPER) was conducted to evaluate within‐ and between‐group variation in FA composition among issue types. Overall, dissimilarity within tissue groups was low (Figure 7), though variation differed by tissue type. Outer‐caudal fin tissue exhibited the highest internal dissimilarity of all tissue groups (average squared distance = 15.84), suggesting a more heterogeneous FA composition across samples. Muscle and inner‐caudal fin tissues displayed moderate within‐group dissimilarity (average squared distances of 12.02 and 10.80, respectively), while adipose fin tissue displayed the most uniform composition (average squared distance = 7.83; Figure 7). Intra‐tissue dissimilarity in outer‐caudal samples was primarily driven by FA18:1n7 (21.50%), whereas dissimilarity within inner‐caudal fin tissue was driven by FAs 20:3n3 (11.49%), 20:0 (10.48%), and 17:0 (10.18%). Within adipose fin tissue, 20:3n3 contributed most to variation (14.78%), while muscle tissue variation was driven by 22:1n11 (30.66%).

Between‐tissue comparisons exhibited markedly greater dissimilarity than within‐tissue comparisons, reflecting the distinct FA composition associated with each tissue type. The greatest compositional difference was observed between outer‐caudal fin and muscle tissues (average squared distance = 130.16), with most FAs contributing relatively equally to this dissimilarity (Figure 7). Adipose fin and muscle tissues displayed the least dissimilarity, with contributions from 14:1n5 and 22:1n11 contributing 15.66% and 14.56%, respectively. Inner‐caudal fin and muscle tissues showed moderate dissimilarity, also driven largely by 14:1n5 (11.87%) and 22:1n11 (11.04%). Outer‐caudal versus adipose fin and outer‐caudal versus inner‐caudal fin comparisons revealed broadly distributed contributions from FAs, indicating no single dominant driver of difference. Dissimilarity between inner‐caudal and adipose fin tissues was driven by 17:0 (11.24%), 15.0 (11.07%), and 20:3n3 (10.87%).

**FIGURE 8:**
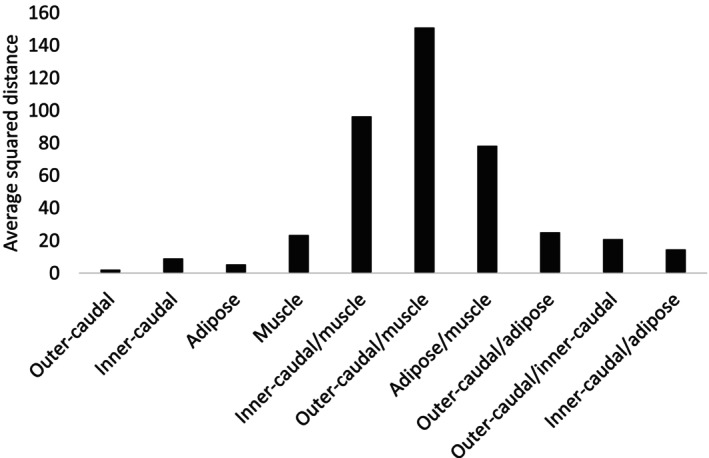
Within group average squared distance for each tissue, based on fatty acid abundance (g/100 g), using Euclidean distance, as calculated using similarity percentage analysis.

A SIMPER analysis, based on FA concentrations (g/100 g), similarly identified both within‐ and between‐tissue differences (Figure 8). While within‐tissue dissimilarity of the outer caudal fin was relatively large for compositional (%‐based) data, it was small when based on concentration (average squared distance = 2.06). Adipose fin tissue (average squared distance = 5.28) and inner‐caudal fin tissue (average squared distances = 9.01) both presented greater amounts of dissimilarity. In contrast, muscle tissue showed the greatest variability (average squared distance = 23.35; Figure 8). In outer‐caudal tissue, C24:1n9 accounted for 58.37% of the observed dissimilarity. This FA also drove variation within inner‐caudal fin tissue (15.19%), while dissimilarity within adipose fin tissue was driven by both 20:4n6 (11.94%) and 24:1n9 (15.88%). For muscle tissue, 14:1n5 and 22:2n6 were primary contributors (11.36% and 11.94%; respectively).

As with composition, the greatest dissimilarity in FA concentrations was found between outer‐caudal fin and muscle tissues (average squared distance = 150.94), with most FAs contributing relatively equally. Comparisons between inner‐caudal fin and muscle tissues (average squared distance = 96.31) and adipose fin and muscle tissues (average squared distance = 78.27) also exhibited high dissimilarity. The most similar tissues were inner‐caudal and adipose fins (average squared distance = 14.67), with dissimilarity primarily driven by 24:1n9 (14.76%) and 20:4n6 (10.99%). Comparisons between outer‐caudal and adipose fin tissues showed lower levels of dissimilarity, driven mainly by 24:1n9 and 20:5n3 (8.26% and 7.96%, respectively). Outer‐caudal and inner‐caudal fin tissues also showed low levels of dissimilarity driven by 24:1n9 (11.23%) (Figure 8).

### 3.5 Linear Regression

**FIGURE 9:**
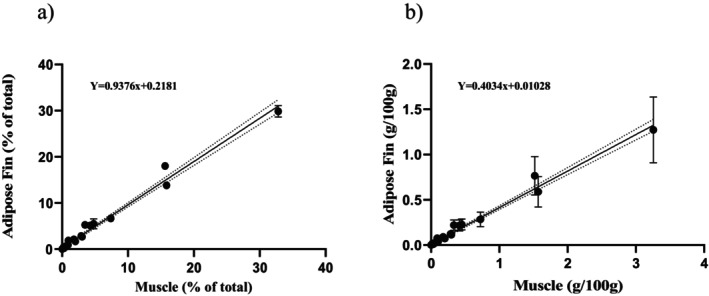
Relationship between adipose fin and muscle tissue fatty acids for: (a) concentrations (% of total) and (b) abundance (g/100 g). The solid line represents the line of best fit and the dotted lines represent the 95% confidence interval (% of total – slope = 0.8912 to 0.9839; Y‐intercept = −0.1384 to 0.5745; and abundance—slope = 0.3807 to 0.4225; Y‐intercept = −0.0068 to 0.0285). Each dot represents a single fatty acid and error bars representing standard deviation.

**FIGURE 10:**
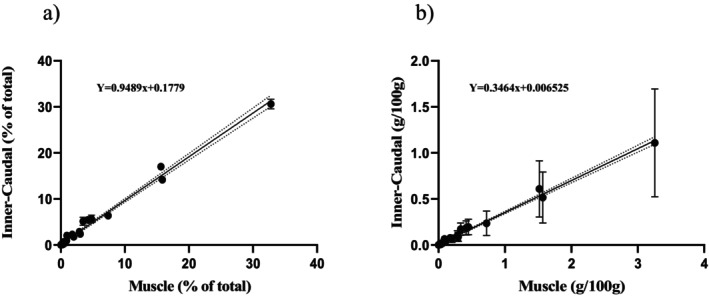
Relationship between inner‐caudal and muscle tissue fatty acids for: (a) concentrations (% of total) and (b) abundance (g/100 g). The solid line represents the line of best fit and the dotted lines represent the 95% confidence interval (% of total—slope = 0.9051 to 0.9876; Y‐intercept = −0.1309 to 0.5035; and abundance—slope = 0.3044 to 0.3309; Y‐intercept = −0.0042 to 0.0181). Each dot represents a single fatty acid and error bars representing standard deviation.

**FIGURE 11:**
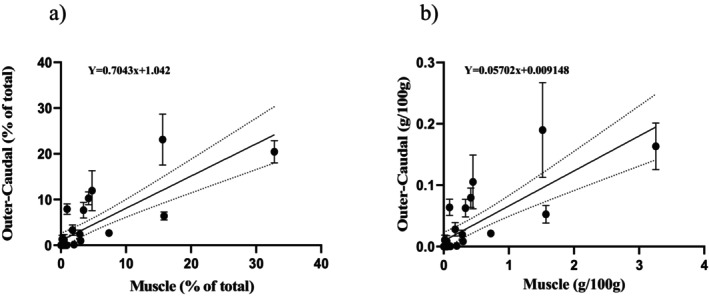
Relationship between outer‐caudal and muscle tissue fatty acids for: (a) concentrations (% of total) and (b) abundance (g/100 g). The solid line represents the line of best fit and the dotted lines represent the 95% confidence interval (% of total—slope = 0.4955 to 0.9075; Y‐intercept = −0.5350 to 2.634; and abundance—slope = 0.3050 to 0.0674; Y‐intercept = −0.0044 to 0.0230). Each dot represents a single fatty acid and error bars representing standard deviation.

Linear regressions were conducted to assess the relationship between FA profiles in muscle tissue and those in the other three tissue types, using both composition (% of total) and concentration (g/100 g) data (Figures 9, 10, 11). Adipose fin tissue showed a strong and highly significant correlation with muscle tissue for both FA composition (*R* ^2^ = 0.98, *p* < 0.001) and concentration (*R* ^2^ = 0.98, *p* < 0.001; Figure 9). Similarly, inner‐caudal fin tissue exhibited a very strong linear relationship with muscle tissue for both FA composition (*R* ^2^ = 0.99, *p* < 0.001) and concentration (*R* ^2^ = 0.99, *p* < 0.001; Figure 10), indicating consistent FA patterns between these tissues. In contrast, outer‐caudal fin tissue exhibited weaker, though still significant, correlations with muscle tissue. For FA composition, the relationship had an *R* ^2^ = 0.64 (*p* < 0.001), while for concentration it was *R* ^2^ = 0.62 (*p* < 0.001; Figure 11).

## 4 Discussion

The goal of this study was to further investigate fish tissues that can be sampled non‐lethally and exhibit a FA profile representative of muscle tissue, enabling the use of FAs as biomarkers in ecological studies. This study identified strong relationships in both FA composition and concentration between muscle and fin tissues (i.e., adipose, inner‐caudal, and outer‐caudal) in Atlantic salmon. The results indicated that adipose fin tissue is a promising candidate as a FA biomarker in salmonids. Additionally, outer‐caudal fin tissue represents a promising non‐lethal sampling option for FA analysis across a broad range of finned fish species.

Importantly, the different analytical approaches used in this study evaluated complementary aspects of tissue similarity rather than redundancy among methods. Linear regressions assessed proportional relationships between tissues across FAs, whereas multivariate approaches such as PCA, PERMANOVA, and SIMPER evaluated overall compositional structure and multivariate dissimilarity. Consequently, tissues could exhibit strong linear relationships with muscle tissue while still differing significantly in multivariate composition. This distinction is particularly important when evaluating non‐lethal sampling methods, as proportional predictability and compositional equivalency do not necessarily represent the same biological phenomenon. The integration of both proportional and distance‐based approaches therefore provides a more comprehensive assessment of tissue suitability for non‐lethal FA analyses.

Among all tissues assessed, adipose fin tissue emerged as the most consistent non‐lethal alternative for FA profiling. Principal component analysis, Euclidean distance metrics, PERMANOVA, and SIMPER analyses collectively suggested strong overlap, low within‐tissue variability, and compositional consistency between adipose fin and muscle tissue. Linear regression analyses further demonstrated strong proportional relationships for both FA composition and concentration (*R* ^2^ > 0.90). These findings further support the interpretation of non‐lethal tissues as functionally informative alternatives rather than exact compositional substitutes.

The lower within‐tissue variability observed in adipose fin tissue relative to muscle tissue may reflect differences in tissue‐specific lipid storage and metabolic dynamics. Muscle tissue integrates both structural and energetic lipid pools and may therefore exhibit greater inter‐individual variation in FA composition and concentration. In contrast, the adipose fin may function as a more compositionally stable lipid reservoir, resulting in stronger proportional consistency among individuals. Additionally, Nanton et al. (2007) reported greater lipid accumulation in the anterior regions of Atlantic salmon relative to posterior regions, further supporting the possibility of spatial variation in lipid storage and FA composition among tissues.

These findings build upon the growing body of research supporting the use of non‐lethal sampling techniques in fish ecology and conservation (Cooke et al. 2017; Jeffries et al. 2021; Olsen et al. 2013; Madhun et al. 2017). The strong proportional relationships, low variability, and relative compositional consistency observed in adipose fin tissue support its potential utility for repeated, minimally invasive sampling.

Although inner‐caudal fin tissue cannot be sampled non‐lethally, its proximity to muscle tissue translated into a strong compositional association. However, compared to adipose fin, the inner‐caudal fin tissue showed more variability and more pronounced differences in FA profiles. Its inclusion in this study provides important insight into intra‐fin variability and highlights the importance of standardized sampling protocols, particularly when working with structurally heterogeneous fin tissues.

Despite its greater compositional variability, the outer‐caudal fin tissue exhibited a significant correlation with muscle tissue (*R* ^2^ = 0.64 for FA composition, *R* ^2^ = 0.62 for concentration). Although these correlations are lower than those observed for adipose or inner‐caudal fin tissues, they are still greater than the typical threshold range (*R* ^2^ between 0.20 and 0.50) considered acceptable for many ecological studies (Lin and Kerstin Wiegand 2023). Given its accessibility and suitability for non‐lethal sampling, the outer‐caudal fin may provide a potential non‐lethal tissue for FA‐based studies, particularly for species lacking an adipose fin. The potential to extend these methods to a broad suite of finned fish enhances the generalizability and conservation impact of our findings; however, the use of this methodology on additional fish species requires additional investigation.

The intra‐fin variability along the caudal fin is attributable to differences in tissue composition, ranging from muscular lipid rich base regions (Schneider and Sulner 2006; Aursand et al. 1994) to collagen‐ and bone‐rich fin rays (Flammang 2014). This structural heterogeneity introduces the potential for intra‐fin variation in FA profiles, which has also been confirmed by both the present study and previous isotopic research by Hayden et al. (2015), which revealed distinct isotopic ratios between the tip and the base of Atlantic salmon fins. These findings reinforce the importance of precise and consistent sampling locations, especially when analyzing tissues that differ markedly from muscle in their structure and biochemical properties.

Despite the growing recognition of non‐lethal sampling approaches in ecological research, relatively few studies have specifically evaluated non‐lethal FA profiling in fish (Jeffries et al. 2021). While previous work demonstrated the utility of adipose fin tissue for non‐lethal FA analyses in Atlantic salmon (Olsen et al. 2013; Madhun et al. 2017), comparisons among tissues based on both FA composition and concentration remain limited, and the application of caudal fin tissue for FA profiling has remained largely unexplored. Additionally, relatively little is known about FA turnover rates among fin and muscle tissues compared to the more extensively studied isotopic turnover dynamics (Larocque et al. 2022; Trueman et al. 2005). Differences in tissue‐specific FA turnover may contribute to the compositional and concentration variability observed among tissues and warrant further investigation. Nevertheless, the strong proportional relationships, low intra‐tissue variability, and relative compositional consistency observed in adipose fin tissue support its utility as a minimally invasive alternative for FA profiling in fish. Furthermore, while outer‐caudal fin tissue exhibited greater variability and weaker relationships with muscle tissue, its accessibility and applicability to species lacking adipose fins suggest that it may provide a promising alternative for non‐lethal FA studies. However, additional validation across species and environmental contexts is needed.

Collectively, these findings broaden the application of FA analyses in conservation physiology, dietary ecology, and long‐term ecological monitoring by reducing the need for lethal sampling. Non‐lethal FA profiling may improve the ability to investigate predator–prey dynamics, migration patterns, food web structure, dietary composition, energetic condition, and environmental responses while minimizing impacts on vulnerable or imperiled fish populations (Lovern 1935; Saddler et al. 1972; Filimonova et al. 2016; Fonseca et al. 2022). Our findings help further address this important knowledge gap and support the application of FA‐based ecological studies in populations where lethal sampling is either impractical or ethically unacceptable, particularly in long‐term or spatially extensive studies requiring repeated sampling or individual tracking.

Several factors should be considered when interpreting these findings. First, this study evaluated a single species originating from a common aquaculture environment with relatively uniform diet and life history. While this controlled for many variables (i.e., diet, age, relative and genetics), it may have reduced the natural variability relative to wild populations. Second, seasonal, environmental, and ontogenetic influences on FA dynamics could not be assessed and may have influenced tissue relationships under natural conditions. Third, tissue‐specific FA turnover rates remain poorly understood and may have contributed to differences among tissues. Additional validation across species, environments, and temporal scales is necessary.

## Author Contributions

**Lauren Comeau:** conceptualization (equal), data curation (equal), formal analysis (equal), methodology (equal), visualization (equal), writing – original draft (lead), writing – review and editing (equal). **Attiq Rehman:** methodology (equal), resources (equal), writing – original draft (supporting), writing – review and editing (equal). **Bruce Phillips:** methodology (equal), resources (equal), writing – original draft (supporting), writing – review and editing (equal). **Jill Hay:** methodology (equal), resources (equal), writing – original draft (supporting), writing – review and editing (equal). **Andrew Swanson:** resources (lead), writing – original draft (supporting), writing – review and editing (equal). **Kurt M. Samways:** conceptualization (equal), data curation (supporting), formal analysis (supporting), funding acquisition (lead), investigation (equal), methodology (supporting), project administration (lead), resources (equal), supervision (lead), validation (supporting), writing – original draft (supporting), writing – review and editing (equal).

## Funding

This work was supported by the Government of Canada Natural Science and Engineering Council of Canada Discovery Grant (RGPIN‐2022‐04466) awarded to Kurt M. Samways.

## Conflicts of Interest

The authors declare no conflicts of interest.

## Data Availability

The data that support the findings of this study are openly available in UNB Dataverse Repository at https://doi.org/10.25545/BWFU2Z.
